# Initial Dip in Estimated Glomerular Filtration Rate After Dapagliflozin Affects Renal Function in Chronic Phase in Chronic Heart Failure

**DOI:** 10.3390/jcm14155246

**Published:** 2025-07-24

**Authors:** Raisa Ogata, Takato Kotaki, Kozue Tanaka, Kyoko Higuchi, Natsumi Kumano, Kyoji Furukawa, Yoshihiro Fukumoto

**Affiliations:** 1 Department of Pharmacy, Kurume University Hospital, Kurume 830-0011, Japan; ogata_raisa@kurume-u.ac.jp (R.O.); koutaki_takato@kurume-u.ac.jp (T.K.); nagaoka_kodue@kurume-u.ac.jp (K.T.); higuchi_kyouko@kurume-u.ac.jp (K.H.); 2 Biostatistics Center, Kurume University, Kurume 830-0011, Japan; a225gm005k@std.kurume-u.ac.jp (N.K.); furukawa_kyoji@med.kurume-u.ac.jp (K.F.); 3 Division of Cardiovascular Medicine, Department of Internal Medicine, Kurume University School of Medicine, Kurume 830-0011, Japan

**Keywords:** dapagliflozin, eGFR initial dip, older age, hypertension, diabetes mellitus

## Abstract

**Background**: Dapagliflozin, a sodium–glucose cotransporter 2 (SGLT2) inhibitor, has been shown to improve prognosis in patients with chronic heart failure (CHF), in whom a transient decline in the estimated glomerular filtration rate (eGFR), known as the “initial dip,” is often observed within the first 1–2 weeks of SGLT2 inhibitor therapy. This study aimed to investigate the factors associated with this initial dip and its impact on long-term renal function in patients with CHF initiating dapagliflozin. **Methods and Results**: This retrospective study included 123 consecutive CHF patients who were started on dapagliflozin at our institution. The presence of an initial dip was defined as a decrease in the eGFR of ≥5 mL/min/1.73 m^2^ within two weeks of initiating therapy. Baseline clinical characteristics and renal function data were analyzed. Older age, hypertension, diabetes mellitus, and a higher baseline eGFR were identified as significant risk factors for the initial dip. Furthermore, both age and the presence of an initial dip were significantly associated with changes in the eGFR at 6 months and 1 year. In patients who experienced an initial dip, the eGFR showed a persistent downward trajectory from the baseline over time. **Conclusions**: An initial dip is more likely to occur in older patients and those with hypertension and/or diabetes mellitus. The presence of an initial dip may also influence long-term renal outcomes and could serve as an indicator of long-term renoprotective efficacy.

## 1. Introduction

Chronic heart failure is a leading cause of hospitalization, especially among individuals aged 65 years and older [1,2,3]. Heart failure is categorized according to the left ventricular ejection fraction (LVEF) into three subtypes, namely heart failure with a reduced ejection fraction (HFrEF; LVEF < 40%), heart failure with a mildly reduced ejection fraction (HFmrEF; LVEF 40–49%), and heart failure with a preserved ejection fraction (HFpEF; LVEF ≥ 50%) [4,5,6]. Approximately half of all patients with heart failure present with HFmrEF or HFpEF, for which effective pharmacological therapies to improve clinical outcomes remain limited [6,7].

Dapagliflozin is a selective oral sodium–glucose cotransporter 2 (SGLT2) inhibitor that acts by persistently, competitively, and reversibly inhibiting SGLT2, a key mediator of renal glucose reabsorption. Based on the results of the phase III DELIVER trial, dapagliflozin has been approved for the treatment of chronic heart failure irrespective of the LVEF [8,9]. In addition to its osmotic diuretic and hemodynamic effects, emerging evidence suggests that dapagliflozin may exert secondary cardioprotective effects, including attenuation of myocardial fibrosis [10].

Moreover, dapagliflozin has been approved for the treatment of chronic kidney disease (CKD) based on findings from the phase III DAPA-CKD trial [11]. SGLT2 inhibition increases distal tubular sodium delivery, enhancing tubuloglomerular feedback and consequently reducing intraglomerular pressure [12]. The resulting hemodynamic benefits—such as corrected fluid overload, blood pressure reduction, and decreased cardiac preload and afterload—are believed to improve renal perfusion and confer renoprotective effects [13].

Long-term administration of SGLT2 inhibitors has been shown to slow the decline in renal function. However, a transient decrease in the estimated glomerular filtration rate (eGFR), known as the initial dip, typically occurs within the first 1–2 weeks of therapy initiation [14,15,16]. This phenomenon is thought to reflect hemodynamic changes within the glomerulus, particularly reductions in intraglomerular pressure. In patients with pre-existing renal impairment, the initial dip may be associated with an increased risk of acute adverse events.

While several studies have investigated the determinants and prognostic implications of the initial dip in patients with chronic kidney disease or diabetes [16,17], evidence remains limited in populations with chronic heart failure. Moreover, few studies have comprehensively assessed the clinical characteristics and contributing factors associated with the initial dip in this patient population. In our cardiovascular department, we have encountered patients with chronic heart failure who exhibited an initial dip in renal function following the initiation of dapagliflozin, often requiring clinical interventions such as the discontinuation of concomitant medications or adjustments to fluid management. In contrast, other patients did not experience an initial dip; however, the comparative trajectory of renal function between these groups remains unclear. Previous studies [16,17] have evaluated long-term renal function using the post-initial dip values as the baseline. However, it is rare for renal function to return to its original baseline once it has declined due to the initial dip. We believe that an accurate assessment of the long-term impact of the initial dip on renal function requires using the pre-initial dip baseline as a reference. To our knowledge, no prior studies have taken this approach, and such a novel perspective may provide new insights into favorable renal trajectories following SGLT2 inhibitor initiation. Identifying factors associated with the initial dip may facilitate a safer and more effective initiation of SGLT2 inhibitors. Furthermore, predicting long-term renal outcomes based on the presence or absence of the initial dip could support individualized treatment planning and enable timely clinical decision-making.

Therefore, this study aimed to investigate the trajectory of renal function following the initiation of dapagliflozin in patients with chronic heart failure. Specifically, we sought to identify factors associated with the occurrence of the initial dip, as well as those predictive of long-term changes in renal function. In addition, we evaluated the long-term impact of the initial dip on renal function using the pre-initial dip baseline as a reference point.

## 2. Materials and Methods

### 2.1. Study Design

This retrospective study included all patients with chronic heart failure who were hospitalized in the Department of Cardiovascular Medicine at our institution and initiated treatment with dapagliflozin 10 mg/day between 27 November 2020 (the date dapagliflozin received regulatory approval for the treatment of chronic heart failure in Japan) and 1 May 2023. Patients were excluded if they had a prior history of SGLT2 inhibitor use, including dapagliflozin, or if they received a reduced dose of dapagliflozin (<10 mg/day), including after treatment initiation.

The present study received ethical approval from the Ethics Committee of Kurume University Hospital (approval no. 23156, 21 November 2023). Informed consent was waived due to the retrospective nature, and opt-out was used in the study. The study was conducted following the ethical principles outlined in the Declaration of Helsinki.

### 2.2. Data Collection

Patient data were retrospectively extracted from electronic medical records. Demographic and clinical parameters at the time of dapagliflozin initiation were collected, including age, sex, height, and body weight. Body mass index was calculated as weight (kilograms) divided by the height squared (square meters). Blood samples were obtained from the antecubital vein and analyzed at a commercial laboratory within Kurume University Hospital to measure the following parameters: aspartate aminotransferase (AST), alanine aminotransferase (ALT), albumin, blood urea nitrogen (BUN), estimated glomerular filtration rate (eGFR), uric acid, blood glucose, sodium, potassium, C-reactive protein (CRP), comorbidities, and concomitant medications. Chronic kidney disease was defined as an eGFR of <60 mL/min/1.73 m^2^.

In addition to the baseline eGFR (Before SGLT2 inhibitor), we also assessed the lowest eGFR value within two weeks of dapagliflozin initiation (acute phase), as well as the values at 6 months and 1 year after treatment initiation.

### 2.3. Definition of Initial Dip of eGFR

Based on previous reports [11,17,18], the initial dip in the eGFR has been estimated to be approximately 3 to 8 mL/min/1.73 m^2^. Therefore, we defined the initial dip as a decrease in the eGFR of ≥5 mL/min/1.73 m^2^ from baseline to the lowest value recorded within two weeks after initiating dapagliflozin. Patients were classified into the “initial dip group” (ΔeGFR ≥ 5 mL/min/1.73 m^2^) or the “non-initial dip group” (ΔeGFR < 5 mL/min/1.73 m^2^).

### 2.4. Outcome

The primary endpoint was to explore factors influencing the initial dip in the eGFR, along with baseline clinical characteristics. Secondary endpoints were to evaluate long-term renal function and changes in the eGFR at 6 months and 1 year after dapagliflozin initiation between the initial dip group and the non-initial dip group.

### 2.5. Statistical Analysis

Continuous variables were summarized as means with standard deviations (SDs), and categorical variables were expressed as counts and percentages. Group comparisons between patients with and without the initial dip were performed using Fisher’s exact test for categorical variables and Student’s t-test for continuous variables. To identify factors associated with the occurrence of the initial dip, univariate logistic regression analyses were first conducted. Variables found to be statistically significant in the univariate analysis were then included in a multivariate logistic regression model. To avoid multicollinearity, variables known to be correlated with the eGFR—specifically CKD, uric acid, and potassium levels—were excluded. Instead, clinically relevant factors presumed to influence changes in renal function were selected for inclusion in the multivariate model. The goodness-of-fit of the logistic regression model was assessed using the Hosmer–Lemeshow test. To evaluate the impact of the initial dip on long-term renal function, multivariate linear regression analyses were performed using the change in the eGFR at six months and one year as dependent variables. Independent variables included the presence or absence of the initial dip, as well as baseline characteristics found to be significant in univariate analyses. Changes in the eGFR were calculated by subtracting the baseline eGFR from the eGFR measured at each respective follow-up time point.

Multiple imputation was used to handle missing data for baseline values for uric acid and electrolytes under the assumption of missing at random (MAR) in the multivariate analysis. All statistical tests were two-sided, with a significance level of 10% for univariate analyses and 5% for multivariate analyses.

All statistical analyses were performed using R Studio version 4.2.1 (Posit PBC, Boston, MA, USA).

## 3. Results

### 3.1. Patient Characteristics

Among the 123 patients who initiated dapagliflozin therapy, 65 patients (52.8%) were classified into the initial dip group and 58 patients (47.2%) into the non-initial dip group. The mean age was higher in the initial dip group (70.5 years) compared to the non-initial dip group (65.3 years) (*p* = 0.07). The baseline eGFR was also higher in the initial dip group (59.4 mL/min/1.73 m^2^) than in the non-initial dip group (44.7 mL/min/1.73 m^2^) (*p* < 0.01). Patients in the initial dip group had a higher prevalence of comorbid hypertension and diabetes mellitus (*p* = 0.02 and 0.03, respectively). Although the use of medications potentially associated with the initial dip—such as angiotensin-converting enzyme (ACE) inhibitors, angiotensin II receptor blockers (ARB), and diuretics—was observed in both groups, no significant differences in concomitant medication use were found between the groups (Table 1).

### 3.2. Risk Factors for Initial Dip

In the multivariate analysis of factors associated with the initial dip, both age and the baseline eGFR at the time of dapagliflozin initiation were significantly associated with the development of an initial dip (*p* < 0.01 for both). Higher age and a higher baseline eGFR were correlated with an increased risk of experiencing an initial dip. Additionally, the presence of hypertension (odds ratio [OR]: 3.24 95% confidence interval [CI]: 1.18–8.95; *p* = 0.02) and diabetes mellitus (OR: 2.61; 95% CI: 1.09–6.25; *p* = 0.03) were identified as significant contributing factors to the occurrence of an initial dip (Table 2). The goodness-of-fit of the logistic regression model was evaluated using the Hosmer–Lemeshow test, which showed no statistically significant difference (χ^2^ = 9.65, df = 8, *p* = 0.29).

### 3.3. Risk Factors Affecting Long-Term Renal Function

eGFR values were available for 77 patients at 6 months and for 62 patients at 1 year; 56 patients had data available at both time points. In the analysis of factors associated with eGFR change at 6 months, both age and the presence of an initial dip were significantly associated with a decline in renal function (*p* < 0.01) (Table 3). Similarly, at 1 year, both age and the initial dip remained significant factors influencing eGFR change (*p* < 0.01) (Table 4). The adjusted R^2^ values of the linear regression models used to identify risk factors associated with changes in the eGFR at 6 months and 12 months were 0.37 and 0.34, respectively.

**Figure 1:**
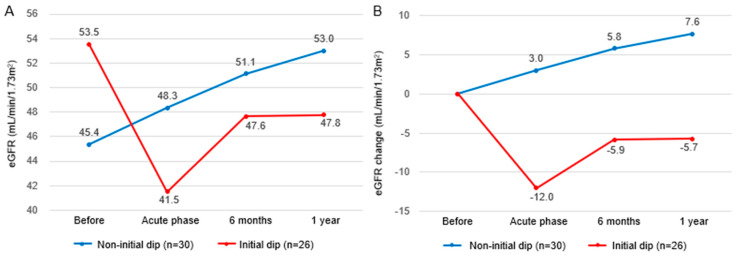
Long-term trajectory of the eGFR according to the presence or absence of an initial dip. (**A**) Changes in absolute eGFR values over time. (**B**) Changes in the eGFR from baseline, defined as the value immediately prior to dapagliflozin initiation. The acute phase is defined as within 2 weeks after initiation.

Figure 1 illustrates the trajectory of the mean values of the eGFR (panel A) and change in the eGFR (panel B) at baseline, the initial dip time point, 6 months, and 1 year in the 56 patients with complete follow-up data. The non-initial dip group showed a trend toward increasing the eGFR over time, whereas the initial dip group did not recover to baseline eGFR values at either 6 months or 1 year, with the eGFR persistently declining.

**Figure 2:**
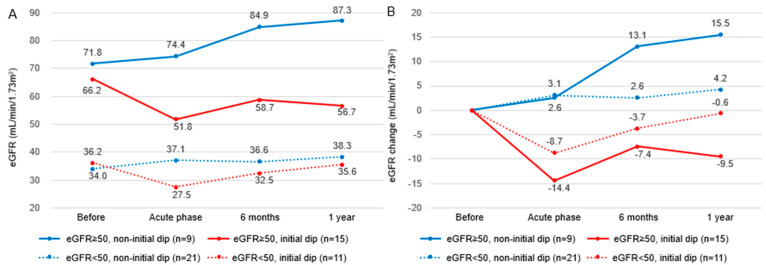
Impact of initial dip on long-term eGFR trajectory stratified by baseline renal function. (**A**) Changes in absolute eGFR values over time. (**B**) Changes in the eGFR from baseline, defined as the value immediately prior to dapagliflozin initiation. The acute phase is defined as within 2 weeks after initiation.

Figure 2 shows the mean values of the eGFR (panel A) and the corresponding changes (panel B) stratified by renal function status (normal renal function: eGFR ≥ 50 mL/min/1.73 m^2^; impaired renal function: eGFR < 50 mL/min/1.73 m^2^) and by the presence or absence of an initial dip. Notably, in patients with eGFR ≥ 50 mL/min/1.73 m^2^ who experienced an initial dip, the mean change in the eGFR was −7.4 mL/min/1.73 m^2^ at 6 months and −9.5 mL/min/1.73 m^2^ at one year, indicating a sustained decline without recovery to baseline levels.

## 4. Discussion

To our knowledge, this is the first study to demonstrate that older age, the presence of diabetes mellitus and/or hypertension, and a higher baseline eGFR prior to initiating dapagliflozin were associated with an increased likelihood of developing an initial dip in the eGFR in chronic heart failure. Previous reports have identified several risk factors for the initial dip, including advanced age, elevated body mass index, higher systolic blood pressure, low hemoglobin levels, smoking, and the use of diuretics [17]. Additionally, the baseline use of diuretics and higher Kidney Disease: Improving Global Outcomes (KDIGO) risk categories have also been associated with the initial dip [19]. Our findings are consistent with previous studies in that older age and comorbid hypertension were significant contributing factors.

In older individuals, sarcopenia and increased adiposity result in reduced lean body mass and total body water, which may contribute to latent renal dysfunction. Although individual variation exists, the eGFR typically declines with age, independent of underlying kidney disease. Aging kidneys exhibit reductions in renal mass and cortical thickness, as well as nephron loss and vascular changes such as a luminal narrowing of arterioles, all of which are exacerbated by hypertension or diabetes [20,21]. Given these physiological vulnerabilities, clinicians should consider a cautious initiation of dapagliflozin in elderly patients, ensuring that blood pressure and glycemic control are well-optimized beforehand.

Furthermore, our study demonstrated that the presence of an initial dip was associated with greater declines in the eGFR at both 6 months and 1 year. Among those who experienced an initial dip, a higher baseline eGFR was associated with greater long-term decline. In the setting of chronic heart failure, acute or chronic dysfunction of the heart or kidneys can lead to deterioration of the other organ, a condition referred to as cardiorenal syndrome [22]. Renal function often deteriorates gradually over the course of heart failure. Sustained hypoperfusion may induce inflammatory cytokine production and profibrotic signaling, aggravating endothelial injury and promoting renal impairment. Additionally, activation of the sympathetic nervous system and renin–angiotensin system can further exacerbate glomerular dysfunction [23]. Some reports have suggested that renal impairment during heart failure is more strongly influenced by elevated central venous pressure than by reductions in cardiac output [24]. These findings highlight the need to consider the role of chronic heart failure-related renal dysfunction in the development of the initial dip and long-term renal trajectory following dapagliflozin initiation.

Multiple randomized controlled trials have demonstrated the cardiovascular benefits of SGLT2 inhibitors, including reductions in heart failure hospitalizations and renal composite outcomes, as well as a slowing of eGFR decline [25,26]. However, there is some evidence that the renal efficacy of SGLT2 inhibitors in preventing heart failure progression may diminish in patients with advanced renal dysfunction [26]. Thus, minimizing the initial dip and preserving long-term renal function is crucial to achieving optimal disease control in chronic heart failure.

The DAPA-CKD trial and database studies have shown that SGLT2 inhibitors are not associated with an increased risk of acute kidney injury (AKI) [11,27]. Moreover, several studies have suggested that the initial dip does not adversely affect long-term renal function [16,17,19]. However, these studies typically evaluated renal outcomes using the post-initial dip eGFR as the new baseline. In contrast, our study assessed long-term eGFR changes relative to the original pre-treatment baseline. Since the eGFR in the initial dip group did not return to baseline levels after the early decline, this distinction may explain the greater cumulative eGFR loss compared to those without an initial dip. In clinical practice, a sustained decline in the eGFR can lead to dose adjustments or discontinuation of renally excreted medications, potentially compromising heart failure management. In our study, patients with preserved renal function at baseline (eGFR ≥ 50 mL/min/1.73 m^2^) who developed an initial dip did not experience eGFR recovery at 6 months or 1 year. These findings suggest that avoiding the initial dip is important not only for preserving renal function from baseline but also for ensuring long-term renal stability. Moreover, the presence of an initial dip may serve as a clinical marker of the long-term renoprotective efficacy of SGLT2 inhibitors, indicating a need for further research into early therapeutic strategies for managing patients with an initial dip.

Although concomitant medications were not associated with the initial dip in this study, previous reports have identified loop diuretics as a risk factor for AKI during SGLT2 inhibitor therapy [28]. A subgroup analysis of the DAPA-HF trial evaluated the safety of the concomitant use of dapagliflozin and diuretics in patients with heart failure and a reduced ejection fraction. The analysis demonstrated that patients receiving a high dose of diuretics—defined as more than 40 mg/day of furosemide equivalent—experienced a higher incidence of volume depletion in the dapagliflozin group compared with the placebo group [29]. Similarly, a study assessing the safety of the combined use of SGLT2 inhibitors and diuretics in patients with diabetes mellitus also reported a significantly increased incidence of volume depletion in those receiving high-dose diuretics exceeding 40 mg/day of furosemide equivalent [30]. These findings suggest that special caution should be exercised regarding volume depletion and the potential risk of AKI when SGLT2 inhibitors are used in combination with high-dose diuretics. Patients with heart failure or advanced age are particularly prone to intravascular volume depletion, which can lead to renal hypoperfusion and AKI. The concurrent use of ACE inhibitors, ARBs, and NSAIDs may also increase the risk of volume depletion. In such situations, clinicians should consider withholding or adjusting concomitant medications or delaying dapagliflozin initiation to avoid worsening renal function.

### Limitations

This study has several limitations. First, it was a retrospective analysis based on electronic medical records, and some data were missing. Moreover, due to the small sample size and the retrospective nature of the study, it was not feasible to account for cardiac function or heart failure events at each time point, which may have influenced the observed renal function trajectories. Although the adjusted R^2^ values were approximately 0.3, which indicates only modest explanatory power, this may be due to the retrospective nature of the study and the use of clinical data, which may have involved unmeasured confounding factors and measurement errors. Nonetheless, our findings provide novel insights suggesting that the initial dip may have a significant impact on long-term renal outcomes. Although we employed multiple imputation under the assumption that data were missing at random, sensitivity analyses conducted without imputation yielded consistent results. Second, as a single-center study with a relatively small sample size, the generalizability of our findings may be limited. While the non-initial dip group showed an upward trend in the eGFR over time, such an improvement is rarely observed in patients with heart failure, regardless of SGLT2 inhibitor use. This unexpected finding may be attributable to the small sample size. Future prospective studies with larger cohorts and complete datasets are warranted to validate these observations. Third, conducting a subgroup analysis based on LVEF categories was deemed inappropriate for this study, as the timing of LVEF measurements varied among individual cases. Including such an analysis could compromise the reliability of the data. Therefore, we assessed renal outcomes irrespective of the LVEF in patients diagnosed with chronic heart failure. Fourth, since this study was not designed to evaluate the effects of SGLT2 inhibitors on arrhythmias [31,32,33], we were unable to analyze this aspect. However, we aim to investigate the multifaceted effects of SGLT2 inhibitors in our future research.

## 5. Conclusions

This study suggested that older age, hypertension, and diabetes are risk factors for the development of an initial dip in the eGFR following the initiation of dapagliflozin. In such patients, the careful initiation of therapy with adequate control of blood pressure and glycemia, along with close monitoring of renal function during the early phase of treatment, is warranted. Notably, in patients with preserved renal function at baseline, the occurrence of an initial dip was associated with sustained long-term declines in the eGFR. These findings indicated that the initial dip may serve as a prognostic indicator of long-term renoprotection, and strategies to mitigate its occurrence should be further investigated.

## Figures and Tables

**Table 1:** Patient baseline characteristics.

		Non-Initial Dip	Initial Dip	*p* Value
		(*n* = 58)	(*n* = 65)	
Age (years old)		65.3 (17.6)	70.5 (14.1)	0.07
Sex (female, %)		22 (37.9)	21 (32.3)	0.57
Body mass index (kg/m^2^)		24.2 (4.37)	24.1 (4.86)	0.84
AST (U/L)		30.0 (16.8)	38.0 (67.6)	0.38
ALT (U/L)		30.9 (23.7)	32.4 (28.5)	0.75
Albumin (g/dL)		3.74 (0.70)	3.60 (0.52)	0.26
BUN (mg/dL)		30.5 (16.2)	22.1 (11.2)	<0.01
eGFR (mL/min/1.73 m^2^)		44.7 (22.6)	59.4 (21.3)	<0.01
Uric acid (mg/dL)		7.66 (2.83)	6.74 (1.78)	0.04
Glucose (mg/dL)		136.3 (59.8)	127.4 (49.1)	0.50
Sodium (mmol/L)		138.8 (2.84)	139.5 (4.10)	0.28
Potassium (mmol/L)		4.34 (0.55)	4.15 (0.50)	0.04
CRP (mg/dL)		1.98 (3.71)	1.56 (2.62)	0.50
Comorbidities				
	Hypertension	35 (60.3)	52 (80.0)	0.02
	Diabetes mellitus	26 (44.8)	42 (64.6)	0.03
	Dyslipidemia	25 (43.1)	32 (49.2)	0.59
	Chronic kidney disease	44 (75.9)	35 (53.8)	0.01
	Infection	7 (12.1)	8 (12.3)	1.00
	Immune disease	5 (8.62)	6 (9.23)	1.00
	Infiltrative diseases (sarcoidosis, amyloidosis)	9 (15.5)	6 (9.23)	0.41
	Endocrine diseases	10 (17.2)	11 (16.9)	1.00
Concomitant medications				
	ACE inhibitors	20 (34.5)	29 (44.6)	0.27
	ARB	17 (29.3)	20 (30.8)	1.00
	Thiazide diuretics	1 (1.72)	1 (1.54)	1.00
	Loop diuretics	34 (58.6)	39 (60.0)	1.00
	MRA	42 (72.4)	50 (76.9)	0.68
	V2RA	22 (37.9)	26 (40.0)	0.85
	Statins	26 (44.8)	32 (49.2)	0.72
	Antidiabetic drugs	16 (27.6)	17 (26.2)	1.00
	Immunosuppressants	10 (17.2)	11 (16.9)	1.00
	Antimicrobial agents	9 (15.5)	8 (12.3)	0.61
	NSAIDs	2 (3.45)	4 (6.15)	0.68

Continuous variables are expressed as the mean (standard deviation), categorical variables as N (%). ACE, angiotensin-converting enzyme; ARB, angiotensin II receptor blocker; MRA, mineralocorticoid receptor antagonists; V2RA, vasopressin V2 receptor antagonists; NSAIDs, non-steroidal anti-inflammatory drugs.

**Table 2:** Analysis of factors affecting the initial dip (*n* = 123).

	Univariate Analysis			Multivariate Analysis		
	Odds Ratio	95% CI	*p* Value	Odds Ratio	95% CI	*p* Value
Age (years old)	1.02	1.00, 1.05	0.08	1.06	1.02, 1.09	<0.01
Sex (female, %)	0.78	0.37, 1.64	0.51			
Body mass index (kg/m^2^)	0.99	0.92, 1.07	0.84			
eGFR (mL/min/1.73 m^2^)	1.03	1.01, 1.05	<0.01	1.07	1.04, 1.09	<0.01
CRP (mg/dL)	0.96	0.85, 1.08	0.50			
Hypertension	2.63	1.18, 5.87	0.02	3.24	1.18, 8.95	0.02
Diabetes mellitus	2.25	1.09, 4.04	0.03	2.61	1.09, 6.25	0.03
Dyslipidemia	1.28	0.63, 2.61	0.50			

CI: confidence interval.

**Table 3:** Analysis of factors affecting the change in the eGFR after 6 months (*n* = 77).

	Univariate Analysis			Multivariate Analysis		
	Predicted Value	95% CI	*p* Value	Predicted Value	95% CI	*p* Value
Age (years old)	−0.41	−0.60, −0.23	<0.01	−0.29	−0.47, −0.11	<0.01
Sex (female, %)	−0.22	−7.58, 7.15	0.95			
Body mass index (kg/m^2^)	0.14	−0.54, 0.82	0.68			
Hypertension	−13.12	−20.39, −5.86	<0.01	−4.58	−11.80, 2.63	0.21
Diabetes mellitus	−6.47	−13.23, 0.30	0.06	−2.39	−8.16, 3.39	0.41
Dyslipidemia	−1.28	−8.20, 5.64	0.71			
Chronic kidney disease	−2.28	−9.55, 5.00	0.53			
Initial dip	−14.01	−20.11, −7.91	<0.01	−9.92	−16.01, −3.83	<0.01

CI: confidence interval.

**Table 4:** Analysis of factors affecting the change in the eGFR after 1 year (*n* = 62).

	Univariate Analysis			Multivariate Analysis		
	Predicted Value	95% CI	*p* Value	Predicted Value	95% CI	*p* Value
Age (years old)	−0.47	−0.71, −0.23	<0.01	−0.37	−0.61, −0.13	<0.01
Sex (female, %)	−2.59	−12.33, 7.14	0.60			
Body mass index (kg/m^2^)	−0.31	−1.37, 0.74	0.56			
Hypertension	−13.75	−23.04, −4.46	<0.01	−5.19	−14.30, 3.92	0.26
Diabetes mellitus	−5.14	−13.78, 3.51	0.24			
Dyslipidemia	0.51	−8.36, 9.38	0.91			
Chronic kidney disease	−0.52	−10.11, 9.07	0.91			
Initial dip	−13.98	−21.91, −6.05	<0.01	−10.90	−18.39, −3.42	<0.01

CI: confidence interval.

## Data Availability

The data underlying this article will be shared with others for the purpose of reproducing results or replicating procedures upon reasonable request to the corresponding author, subject to institutional and ethics committee approval.

## Abbreviations

The following abbreviations are used in this manuscript:

SGLT2	sodium–glucose cotransporter 2
CHF	chronic heart failure
eGFR	estimated glomerular filtration rate
HFrEF	heart failure with reduced ejection fraction
HFmrEF	heart failure with mildly reduced ejection fraction
HFpEF	heart failure with preserved ejection fraction
CKD	chronic kidney disease
AST	aspartate aminotransferase
ALT	alanine aminotransferase
BUN	blood urea nitrogen
CRP	C-reactive protein
SDs	standard deviations
MAR	missing at random
ACE	angiotensin-converting enzyme
ARB	angiotensin II receptor blockers
OR	odds ratio
CI	confidence interval
AKI	acute kidney injury
